# In Their Own Words: A Qualitative Study of Kenyan Breast Cancer Survivors’ Knowledge, Experiences, and Attitudes Regarding Breast Cancer Genetics

**DOI:** 10.1200/JGO.17.00061

**Published:** 2017-12-04

**Authors:** Siwon Lee, Amal Gedleh, Jessica A. Hill, Seemi Qaiser, Yvonne Umukunda, Philip Odiyo, Grace Kitonyi, Helen Dimaras

**Affiliations:** **Siwon Lee**, **Amal Gedleh**, **Seemi Qaiser**, **Yvonne Umukunda**, and **Helen Dimaras**, University of Toronto; **Jessica A. Hill** and **Helen Dimaras**, The Hospital for Sick Children; **Helen Dimaras**, SickKids Research Institute, Toronto, Ontario, Canada; **Grace Kitonyi**, and **Helen Dimaras**, University of Nairobi; and **Philip Odiyo**, Faraja Cancer Support Trust, Nairobi, Kenya.

## Abstract

**Introduction:**

Breast cancer ranks among the most common adult cancers in Kenya. Individuals with a family history of the disease are at increased risk. Mutations most commonly associated with breast cancer affect *BRCA1* and *BRCA2*; mutations in several other genes may also confer breast cancer risk. Genetic testing and counseling can help patients understand their risk and assist clinicians in choosing therapies. We aimed to uncover what patients know, experience, and think with regard to breast cancer genetics in Kenya.

**Methods:**

Participants included breast cancer survivors age > 18 years. Participants completed a demographic questionnaire before participating in focus group discussions to uncover knowledge of, experiences with, and attitudes toward the genetics of breast cancer. Data were analyzed by inductive thematic analysis.

**Results:**

Four focus groups were conducted. Participants had rudimentary knowledge about genetics and cancer development, and although they understood breast cancer could be familial, many suspected environmental factors causing spontaneous disease. They reported limited experience with counseling about genetic risk, perceiving that their physicians were too busy to provide comprehensive information. Many indicated they promoted cancer screening among family to promote early diagnosis. Participants expressed a need for more comprehensive counseling and access to genetic testing, recognizing the added clarity it would bring to their families’ risk of cancer.

**Conclusion:**

Improved communication from health care teams could clarify the risk of cancer for affected families. The introduction of affordable genetic testing and counseling for breast cancer in Kenya is welcomed by survivors.

## INTRODUCTION

Breast cancer is the most common cancer among Kenyan women.^1^ African women are generally diagnosed with advanced disease, which requires more intensive therapy and has a reduced chance of survival.^2,3^

Family history of breast cancer increases the risk of developing cancer^4^; knowledge of this risk can facilitate early screening and better outcomes. Hereditary breast cancer is associated with mutations in *BRCA1*^5-8^ or *BRCA2*^9^; mutations in other genes may also confer breast cancer risk.^10^ Clinical genetic testing identifies causative familial mutations. Genetic counseling helps patients understand their risk. However, in low- and middle-income countries, cancer genetic services are often unavailable or inaccessible because of financial and other barriers. Genetic counseling is also limited, because genetic counselors are rare and physicians lack training in this respect.

New technologies have made genetic testing a reality in resource-limited settings.^11,12^ There is a need for concomitant enhancement of genetic counseling. It is equally important to understand what cancer survivors know about the genetics of their disease and uncover their experience with and perspectives on cancer genetics, because use of genetic services is dependent on social factors.^13^ In this respect, we conducted focus groups with Kenyan breast cancer survivors to gain insight into their knowledge, attitudes, and experiences regarding genetics and heritable cancer.

## METHODS

### Study Design

This qualitative, cross-sectional study aimed to uncover Kenyan breast cancer survivors’ knowledge, experiences, and attitudes regarding breast cancer genetics. The study was approved by the University of Nairobi Ethics and Research Committee.

### Participant Recruitment

Participants were recruited from monthly breast cancer support groups hosted by Faraja Cancer Support Trust, a cancer support organization. Interested participants were asked to connect with the study team to enroll in the study.

Kenyan female breast cancer survivors age > 18 years who could communicate in either English or Swahili were eligible. A breast cancer survivor was defined as an individual who had received a breast cancer diagnosis and undergone treatment in Kenya. All participants provided written informed consent.

### Data Collection

Participants completed a brief questionnaire, which asked for basic demographic information and details about their diagnosis (Tables 1 and 2). Focus groups took place at Faraja Cancer Support Trust. Discussions were moderated in English and translated into Swahili in real time as necessary. Discussions lasted 60 to 90 minutes and were audio recorded. The interview guide asked participants to comment on: their understanding of breast cancer and its causes, genetics, and inheritance; primary source(s) of information; primary sources of psychosocial support; understanding of the long-term implication(s) of breast cancer; and challenges faced during their experience.

**Table 1:**
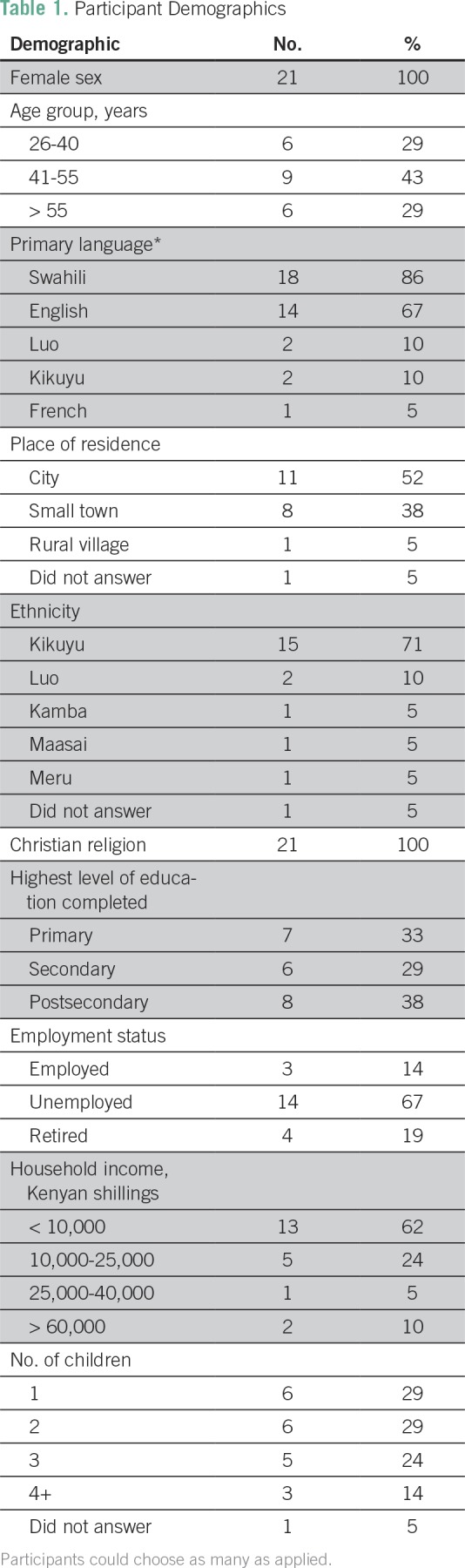
Participant Demographics

**Table 2:**
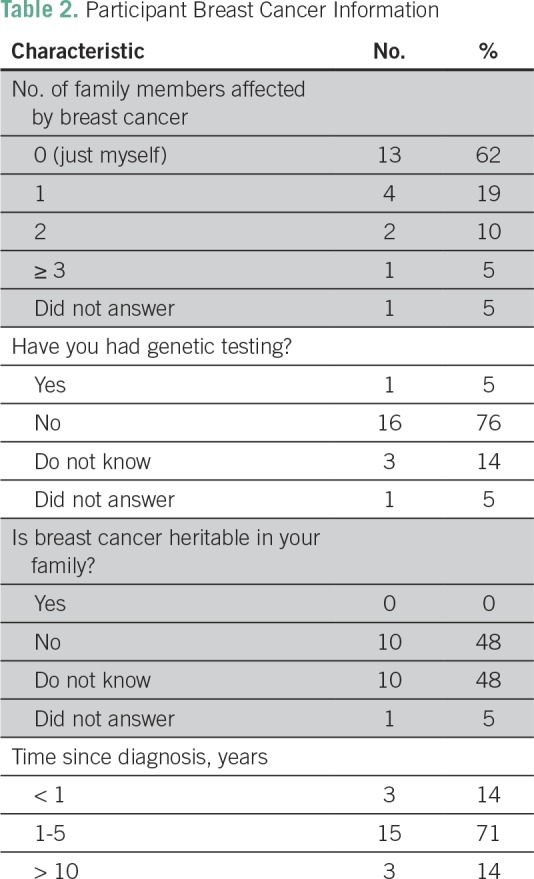
Participant Breast Cancer Information

### Data Analysis

Audio recordings were transcribed verbatim in English. Participants were deidentified using alphanumeric codes (eg, J1). Data were managed using NVivo 11 software (QSR International, Melbourne, Victoria, Australia). Two researchers coded the transcripts independently, using a codebook generated together. Discrepancies were settled by a third researcher. Data were analyzed using inductive thematic analysis.

## RESULTS

### Participant Demographics

Four focus groups with 21 female breast cancer survivors were conducted. Most participants were between the ages of 41 and 55 years (43%), identified Swahili (86%) or English (67%) as a primary language, lived in a city (52%), and had Kikuyu ethnicity (71%). Additional demographic information is listed in Table 1.

Most participants reported being the only person in their family diagnosed with breast cancer (62%). Most were diagnosed in the last 1 to 5 years (71%). One participant reported having undergone genetic testing. When asked if breast cancer was heritable in their family, participants responded either no (48%) or unknown (48%; Table 2). A summary of the themes and subthemes of the study, detailed in the following section, is provided in Table 3.

**Table 3:**
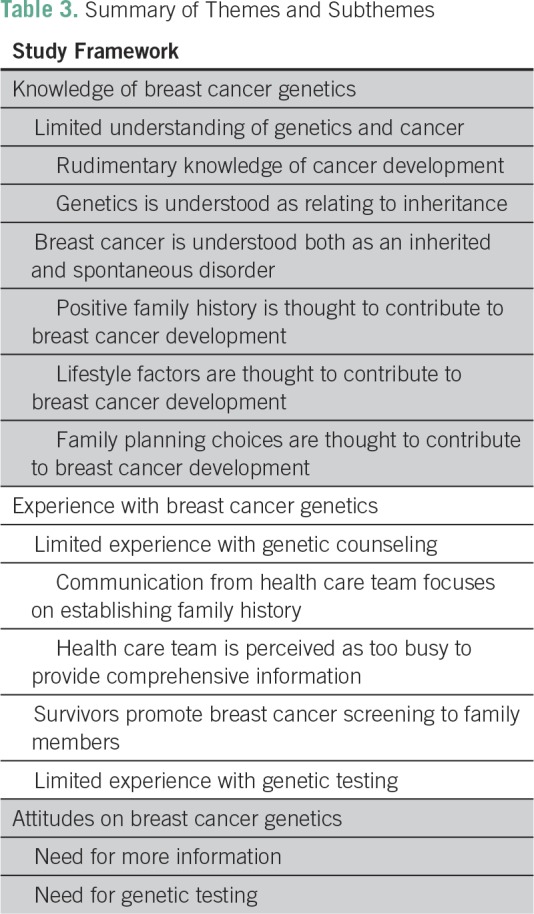
Summary of Themes and Subthemes

### Knowledge of Breast Cancer Genetics

#### Limited understanding of genetics and cancer.

##### Rudimentary knowledge of cancer development.

Participants generally described breast cancer as the proliferation of abnormal cells:

“I would define the word cancer as I have gone through. Normal cells in the body that turn so they become abnormal and they start multiplying. They multiply uncontrollably. Something like that.” —J3

One participant initially thought cancer was a communicable disease because people in her family had different types of cancer:

“I was very sure much worried because my husband died in 1991 because he had a stomach cancer and my sister died in 1996, she had a cervical cancer. Then I thought one of them gave me the cancer.” —M7

##### Genetics is understood as relating to inheritance.

Most participants understood terms genetics or inheritance as relating to familial traits passed on from generation to generation:

“I think the genetic is something to do with genes, the makeup, what makes up the genes that make a person. And when you have now the gene that is having the cancerous, makeup, is the one that makes you a potential for cancer. Now you get it because of … you inherited it from the parents. One of your parents could be carrying the gene and then pass it on to you. And then you can either have it because you get it from both the parents, you can get it from one of them. Or you can maybe lucky and you don’t get it but you still carry it to one of your child or something like that.” —J6

However, some expressed more familiarity with the term inheritance than with gene or genetic:

“I don’t know genetic but inheritance. You inherit from your family. It runs in the family. That’s the meaning of the word inheritance. But I don’t know, I’m not very clear about genetic.” —J5

#### Breast cancer is understood both as an inherited and spontaneous disorder.

##### Positive family history.

When family history was present, participants concluded that the cancer could have been inherited; however, they were not certain:

“I think as for me my mom had breast cancer. And she died when she was just 33 years from breast cancer. When they took the test for my disease, they said it was triple negative, it was not caused by hormones. The doctor say that it could be inherited but I have not done any test to conclude that. Yeah, so I thought that maybe I could have got the gene from my mother.” —M2

##### Lifestyle factors.

Participants often pinpointed specific lifestyle factors that could cause cancer:

“Then of course the regular things that we are told all the time. Smoking, eating the wrong food, not exercising, those things, combination of factors, including stress.” —J4

##### Family planning.

Family planning methods were identified as possible cancer causes. The absence of family history sometimes made it easier to believe birth control may have been the cause:

“Her mom doesn’t have it and she has never heard of breast cancer before. So that’s why she thought family planning might be the cause.” —J22

Birth control as a cause of breast cancer development was perceived to be supported by physicians:

“In her case, she thought it was family planning. She had—I think a patch?—that she used to put on her hand, and she got a lump. And she had been using the family planning for three years. So when she went to see the doctor, the first thing he said was to remove the patch.” —J21

### Experience With Breast Cancer Genetics

#### Limited experience with genetic counseling.

##### Communication from health care team focuses on establishing family history.

Some participants were given some form of risk counseling for family members:

“[The doctor] told me if I have a daughter, since it is running in the family, and my mother died at 64, I got mine, I was diagnosed at 43. So he told me that my daughter should start going for breast cancer examinations at 23. Yeah, half the age.” —J6

Some participants reported not having a discussion on genetics but being interviewed about their family history:

“No, there wasn’t any discussion on that [genetics]. But there is a form that they were filling whereby they were asking you if your dad had cancer or your mom had breast cancer.” —J21

##### Health care team is perceived to be too busy to provide comprehensive information.

Participants believed that health care professionals were too busy to communicate to their families about familial risks of breast cancer:

“They [the doctors] actually don’t have time. Because we would like them to at least spend some time to call our family, maybe our spouse or our children and then maybe they are in a better position to explain to them and then us, ourselves.” —M2

An underlying current of distrust of medical professionals was evident in most focus groups. The business of physicians was often explained as resulting from a perceived financial incentive:

“They don’t want to take their time to explain to you because they are expecting the next patient. Because as many patient gets in that is how they are counting their pocket.” —J5

##### Survivors promote breast cancer screening.

Participants did not explicitly speak about increased risk of cancer to family members. However, they promoted screening to their family:

“So like in my family, I’ve sensitized the people around me, my sisters and my brothers. I’ve encouraged my sisters to be going for mammograms and my mom.” —J1

One participant encouraged her daughter to get a mammogram but recognized the financial barrier to accessing the service:

“I won’t like her to pass through what I passed through…. Because mammogram, she had when she had money. She should go for checkup. So the moment she gets money, I’m encouraging her to go for checkup.” —J14

Participants also spoke about how female family members held the worry that they would also be affected:

“But I remember the first month, my daughter who is 20 now. She was very devastated. She used to Google and find out about breast cancer. She would always touch her breasts to find out if she had a lump.” —J14

#### Limited experience with genetic testing.

Most participants reported having no experience with genetic testing. One individual had accessed genetic testing and counseling in India. The experience was described as having provided clarity:

“For my case, I’m the first one to have cancer in my family, my extended family, from whichever side. I have done a genetic test and it was negative. So I’m happy at least it does not go down to my children.” —J13

### Attitudes on Breast Cancer Genetics

#### Need for more information.

Many expressed a need to better understand breast cancer to develop further competency in informing family members:

“At that point, you don’t even know what breast cancer is. So you just tell them it is breast cancer but they don’t know what it is and you yourself don’t know what it is.” —J13

#### Need for genetic testing.

One participant spoke of the value of genetic testing but stressed that it is inaccessible in Kenya because of high costs:

“When [my sister] learned that I have breast cancer, she lives in the US, she went for that test, the genetic test. She did. Which came out negative. She said it was expensive but it was necessary for her to alleviate the fear and know that. So for me, I think it’s something that people should learn, maybe to embrace. Because like you say, knowledge is power. Because when you know, at least it can be reached by each and everybody, because here in Kenya we want these things to happen but like what she said, sometimes you have nothing. You don’t have any coins.” —J6

Some saw value in providing affordable genetic testing in the future:

“No one ever told me to go for a gene test or…. So I have a daughter. I would not like her to go through what I’ve gone through that by the time they came to diagnose [the cancer] in me, the cancer was at third stage. They could have seen it early, at an early stage so I think if they gave us some affordable gene test, we will take our children, our girl child, and from there they will tell us the way forward.” —M2

## DISCUSSION

The recent focus on and efforts to build capacity in African genomic science promises to revolutionize care for genomic disorders like cancer.^11,12^ This will require strengthening of genetic counseling services; therefore, we sought to uncover how breast cancer survivors in Kenya understand, experience, and conceptualize the underlying genetics of their disease. We found that survivors generally understood that breast cancer could be heritable but did not often fully comprehend how this risk translated to themselves or their family members. The demographic survey revealed that although seven participants knew of family members who also had breast cancer, they reported that breast cancer was not heritable, or they did not know if it was heritable in their case. Again, later in discussions, participants cited positive family history but used terms such as 'maybe' and 'could be' when talking about whether they had inherited the trait; one participant initially thought her cancer was communicable. It is important for health care practitioners to be aware of this misunderstanding so that they can dispel confusion. Participants who indicated they had triple-negative breast cancer (sometimes described as nonhormonal) mentioned it during the discussion of inheritance. Triple-negative breast cancer does not express receptors for estrogen, progesterone, or human epidermal growth factor receptor 2/neu, and can be suggestive of heritable breast cancer, especially in women diagnosed at age < 60 years.^14^ International guidelines recommend that individuals with triple-negative breast cancer be referred for genetic testing or counseling. Triple-negative breast cancer may have a higher incidence in Africa^15^; it is possible that physicians are aware of this risk and discuss the possibility of hereditary disease more comprehensively with these patients.

Participants offered much discussion on environmental or lifestyle factors that are known to increase risk of breast cancer. Survivors often indicated they perceived use of birth control as a risk factor in breast cancer development. Despite this widespread perception, only one study has shown a small increased risk of breast cancer while using contraceptives, which diminishes over time.^16^ More importantly, another study showed robust evidence that contraceptive use confers no increased risk of breast cancer to the general population.^17^ Only for *BRCA1* mutation carriers do oral contraceptives incur this increased risk^18^; however, in Kenya, where genetic testing is not performed routinely, none of the participants in our study would have known if they were *BRCA1* carriers. It is possible that because physicians do not know the mutation status of the patient they are counseling, they may speculate that their patient is a mutation carrier and counsel conservatively. Taken together, the abundance of misinformation about breast cancer illustrates a need for genetic testing and counseling to comprehensively and accurately outline risks to facilitate informed decision making on the part of patients.

Study participants reported limited experience with genetic counseling. Many recalled being asked for detailed family history by their physicians but often perceived that health care professionals were too busy to provide them with quality communication and were mainly interested in generating revenue. The underlying mistrust of physicians prevalent in our discussions is consistent with prior research suggesting that commercialization of medicine significantly weakens patient-physician trust.^19^ In China, commercialization of medicine, along with high patient expectations and inadequate training of physicians in patient communication, is a contributor to patient-physician mistrust, often with violent consequences.^20,21^ In Kenya, further research may be necessary to delineate how medical mistrust may affect uptake and use of genetic services.

Incorporating genetic counseling into standard of care could help improve patient understanding and strengthen the patient-physician relationship. However, there may be an unrealistic patient expectation that physicians have a comprehensive understanding of genetic testing and its implications. In reality, studies from around the world consistently have shown that physicians perceive themselves to be ill versed in clinical genetics or would prefer assistance in sharing results with patients,^22-24^ and even medical oncologists score low on knowledge tests of cancer genomics.^25^ Genetic counselors are primed to take this role^26^; however, in Kenya, where genetic counselors do not exist, alternative models of counseling may need to be developed and tested.^27^ Disease-based genetic education programs for physicians may be one way to build capacity in this area.^28^

Genetic testing could also improve knowledge of and clarify communication from the health care team. There is a growing body of evidence suggesting that all women should be tested for breast cancer–predisposing mutations, regardless of presence or absence of family history.^29,30^ Some might argue that a positive genetic test might cause undue stress; however, evidence indicates that genetic testing and appropriate counseling does not increase distress over time.^31-33^ A systematic review on uptake of genetic services finds that 59% of women with breast cancer choose to undergo genetic testing.^34^ Our participants expressed a desire for access to genetic services, rationalizing that it could help relatives identify their risk of breast cancer. Hereditary breast cancer is also associated with increased risk of other cancers (eg, ovarian),^10^ for which genetic testing would help deduce the risk; however, no participant discussed this additional benefit.

The one participant who had received genetic testing and counseling had traveled to India for treatment, presumably having the financial means to do so. Financial barriers are known to prevent uptake of genetic services,^35,36^ and in our study, this was identified as a recognized challenge to overcome. In future, genetic testing could be covered by the national health insurance fund, as one way to provide affordable service to patients. Physician recommendation is another predictor of uptake,^35,37^ so it is important to educate physicians on this opportunity as it develops in Kenya. It is possible to develop breast cancer genetic services in sub-Saharan Africa.^38-40^ However, given the distrust of the medical community uncovered by our study, the value of genetic testing must be clearly articulated to patients to avoid further damaging patient-physician relations.

The main limitation of this study is that it represents perspectives from survivors from a relatively uniform population that primarily identified as urban, Kikuyu, and having completed secondary school or higher. Additional studies in different geographic areas and demographic groups may yield more diverse perspectives.

In conclusion, there is a need for improved education among Kenyan breast cancer survivors about genetics. With better understanding of genetics through tailored education in support groups, patient-oriented genetic testing and counseling could be introduced in Kenya in the near future.
